# Emerging Insights into European Markets of Biologics, Including Biosimilars

**DOI:** 10.3390/ph15050615

**Published:** 2022-05-17

**Authors:** Steven Simoens, Isabelle Huys

**Affiliations:** Department of Pharmaceutical and Pharmacological Sciences, KU Leuven, 3000 Leuven, Belgium; isabelle.huys@kuleuven.be

Biological medicinal products have revolutionised the treatment of many diseases, e.g., autoimmune diseases and cancer, by targeting key disease mediators with high specificity. As patents and other exclusivity rights on many high-selling and expensive biologics are expiring or have expired, biosimilars may enter the market. The market entry of biosimilars (the first of which was approved in the European Union in 2006) has raised questions about legal, regulatory, pricing and reimbursement procedures for these products, as well as regarding policies and incentive structures related to, for example, tendering mechanisms, gainsharing practices, physician quotas, prescribing and switching frameworks, substitutions and the education of stakeholders. 

In response to this, KU Leuven (Leuven, Belgium), in collaboration with the Erasmus University Medical Center (Rotterdam, the Netherlands), established the MABEL research programme in 2016, with the aim of exploring the market environment of biologics, including biosimilars, in Europe. On the programme’s fifth anniversary, we launched a Special Issue on “Biosimilars in Europe” to share some emerging insights derived from our research programme and from articles published in the Special Issue, as well as to identify unresolved questions and set out a research agenda for the future.

The Need to Reap the Rewards of Biosimilar Competition

The introduction of biosimilars may create competition, possibly resulting in lowered prices, altered market dynamics and the revision of company strategies; it might also attract new players to the biopharmaceutical market. As a result, some health care systems have embraced biosimilars as a tool to control increasing health care expenses or expand patient access to treatments. Competition between off-patent biologics and biosimilars may also induce incremental innovation and the development of next-generation biologics with, for example, a novel formulation or route of administration.

Three articles in this Special Issue provide empirical evidence concerning some of these rewards of biosimilar competition. A Spanish budget impact analysis estimated that biosimilar competition yielded a total saving of EUR 2.3 billion from 2009 to 2019, with approximately one-half of the savings originating from a reduction in list prices and the other half originating from hospital tender discounts [1]. Although total savings over this period were impressive in absolute terms, savings in relative terms amounted to less than 4% of pharmaceutical expenditures in 2019. In an analysis of the Bulgarian market for biologic disease-modifying antirheumatic medicines, Tachkov et al. showed that biosimilar market entry not only reduced prices, but also increased utilization (thus, widening patient access to treatment) and generated competition in a therapeutic class [2]. Finally, a Belgian study examined the introduction of an intravenous biosimilar in the presence of a subcutaneous reference biologic, and indicated that a cost comparison between such products needs to consider multiple factors, such as patient’s body weight, discounts and intravenous vial sharing [3].

The Special Issue also confirms that not all European countries are currently reaping the full rewards of biosimilar market entry and competition. On the one hand, Moorkens et al. suggest that the relatively high market shares of infliximab and etanercept biosimilars in Germany were attained through the implementation of biosimilar prescription quotas, variable procurement contracts between sickness funds and manufacturers, and gainsharing arrangements [4]. On the other hand, Lobo and Río-Álvarez explain how biosimilar competition in Spain is impeded by a variety of barriers, including physician and patient lack of trust in biosimilars and diverging stakeholder interest [5].

The Need to Prescribe Best-Value Biologics

There has been much debate regarding the appropriate use of off-patent reference biologics, biosimilars and next-generation biologics. Instead of promoting the use of one over the other, we believe that the focus needs to shift towards the prescription of best-value biologics. Although the latter term is not uniformly defined, countries such as Ireland and England have implemented programmes stimulating the use of best-value biologics, which may be the off-patent reference biologic, a biosimilar version or a next-generation biologic. By framing the debate in the broader context of best-value biologics, it is possible to align the interests of different stakeholders towards the common objective of maximizing population health with limited resources. However, the introduction of such a programme is not easy, as described in the article by Van Wilder concerning the 2019–2020 “Best-Value Biologics” programme in Belgium [6].

We see an important role for hospital tender procedures to achieve the selection of best-value biologics. Based on a review of tender procedures for off-patent biologics and biosimilars in Europe, Barbier et al. highlighted the importance of creating a level playing field, of timely launching tenders in accordance with public procurement laws, and of guaranteeing supply by creating room for several manufacturers to be active in the market [7]. In addition to the design of tender procedures, competition and incentives were perceived to be crucial in creating a sustainable market for best-value biologics by a panel of European experts [8]. This article makes an important contribution to the field by proposing a consensus definition and identifying some ‘dos and don’ts’ of a competitive, but sustainable, market for off-patent reference biologics, biosimilars and next-generation biologics. However, much more research needs to be carried out to build a comprehensive theoretical framework to understand how European competitive markets of biologics, including biosimilars, can also be sustainable.

When selecting a best-value biologic or in general terms a best-value medicine, there is a need to consider a whole therapeutic class of products. Let us take the example of rheumatoid arthritis. Although there are differences in indications and target populations, the therapeutic arsenal for rheumatoid arthritis consists of synthetic disease-modifying antirheumatic medicines (e.g., methotrexate and leflunomide), off-patent reference biologics and their biosimilars (adalimumab, infliximab and etanercept), other reference biologics (abatacept, golimumab, sarilumab, tocilizumab and certolizumab pegol) and the recent targeted synthetic Janus kinase inhibitors (tofacitinib and baricitinib). As the market entry of biosimilars and novel biologic or synthetic medicines is likely to influence the relative (cost-)effectiveness of products within a therapeutic class, treatment guidelines need to be regularly updated. However, this is not regularly performed, and, in relation to our example, there is a need for research which assesses the value of Janus kinase inhibitors versus all therapeutic alternatives for rheumatoid arthritis.

The Need to Optimise and Harmonise Regulatory Procedures

The European Medicines Agency has been a worldwide frontrunner in developing and implementing a regulatory pathway supporting the marketing authorisation of biosimilars, with the United States, Canada and Japan adopting similar pathways. The article by Ingram et al. presents a unique insight into how the regulatory agencies from these four countries responded to virtually the same set of data on eight candidate biosimilars from one company [9]. Even though authorisation decisions were the same, the authors noted some differences in how the regulatory agencies tackled the data review and benefit–risk assessment. This lack of uniformity may raise the cost of biosimilar development and may also hamper patient access.

At the time of marketing authorisation, the European Medicines Agency publishes an extensive and detailed scientific assessment report (the so-called European Public Assessment Report) concerning all aspects of a medicine. The article by Alsamil et al. evaluated the critical quality attributes in the European Public Assessment Reports of all adalimumab biosimilars, corroborating that these biosimilars have the same functions and clinical profiles, notwithstanding small variations in glycoforms and charge variants [10].

The Need to Educate Patients

Despite all the efforts and existing programmes available, informing and educating patients regarding biosimilars, there remains scepticism towards their use. Indeed, the article by Vandenplas et al. showed that biosimilar information provided by European patient organisations themselves is not always correct or sufficiently detailed [11]. Hence, this paper sends forth a call for regulatory authorities, industry associations, health care professional associations and patient organisations to jointly produce and disseminate unbiased information concerning biosimilars in a language that is accessible for patients. An additional avenue is to develop a dedicated European Commission-driven website for patients (and health care professionals) on biosimilars.

The Need for Further Research

Taking inspiration from Hippocrates, market and policy research of biologics, including biosimilars, should strive to declare the past and diagnose the present, with the intention of foretelling the future. In respect to the latter, additional research is needed, moving beyond identifying hurdles to biosimilar market entry and competition, and analysing the impact of strategies to overcome these hurdles. Furthermore, questions remain concerning the long-term sustainability of European markets of biologics, including biosimilars: how do we create a policy environment that not only promotes competition, but also safeguards economic viability and prevents shortages?

## Data Availability

Not applicable.
